# Supplementary material for: DMRforPairs: identifying Differentially Methylated Regions between unique samples using array based methylation profiles
Source: BMC Bioinformatics. 2014 May 15;15:141. doi: 10.1186/1471-2105-15-141 (PMC4046028; doi:10.1186/1471-2105-15-141)
Supplement: Additional file 2 — DMRforPairs output for the comparison of A431-MCF7 and NA17018-NA17105. Please start from the HTML files in each folder. Available via the BMC Bioinformatics website. [file 1471-2105-15-141-S2.zip › 1394847754114233_MOESM2_ESM/A431_MCF7/figures/35510.pdf]

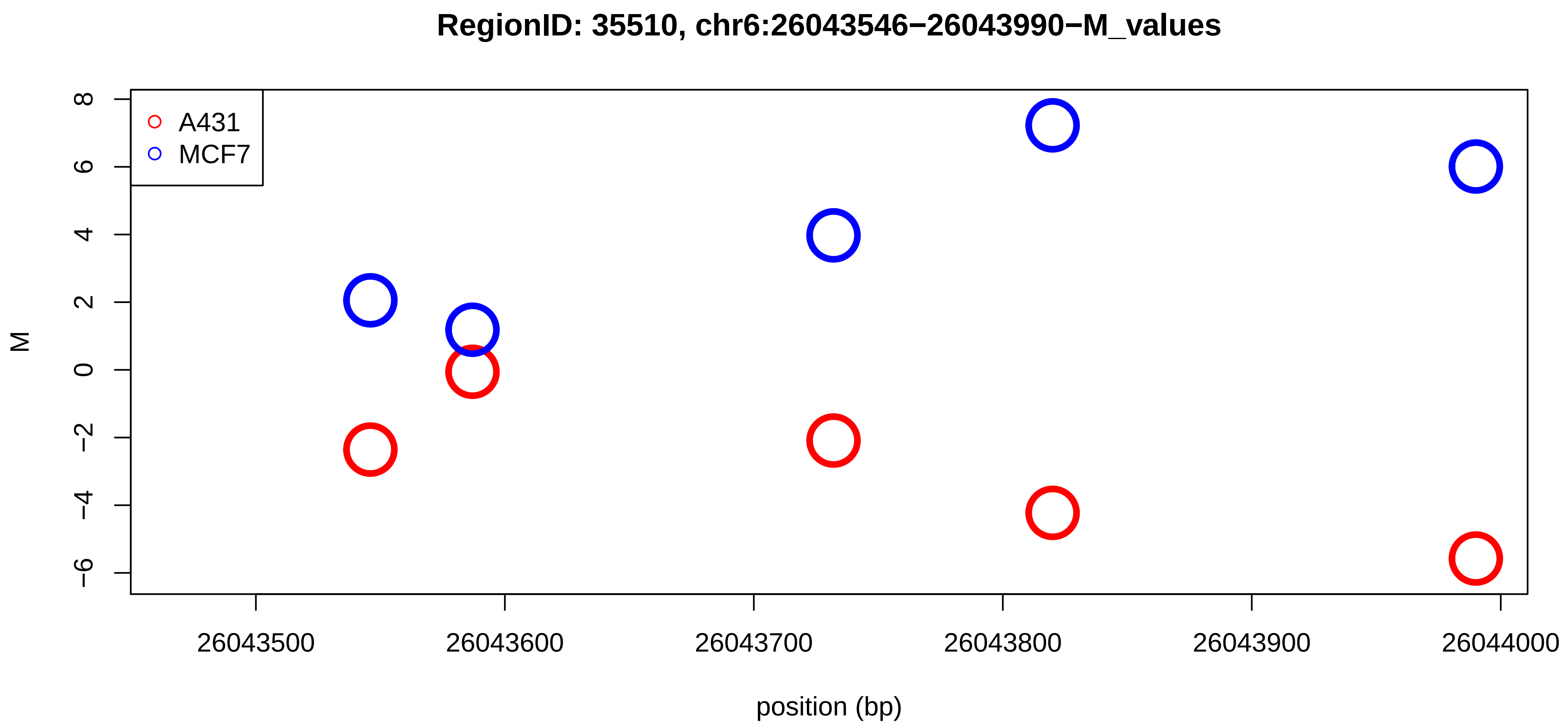
RegionID: 35510, chr6:26043546−26043990−M_values
A431
MCF7
M
8
6
4
2
0
−2
−4
−6
26043500
26043600
26043700
26043800
26043900
26044000
position (bp)

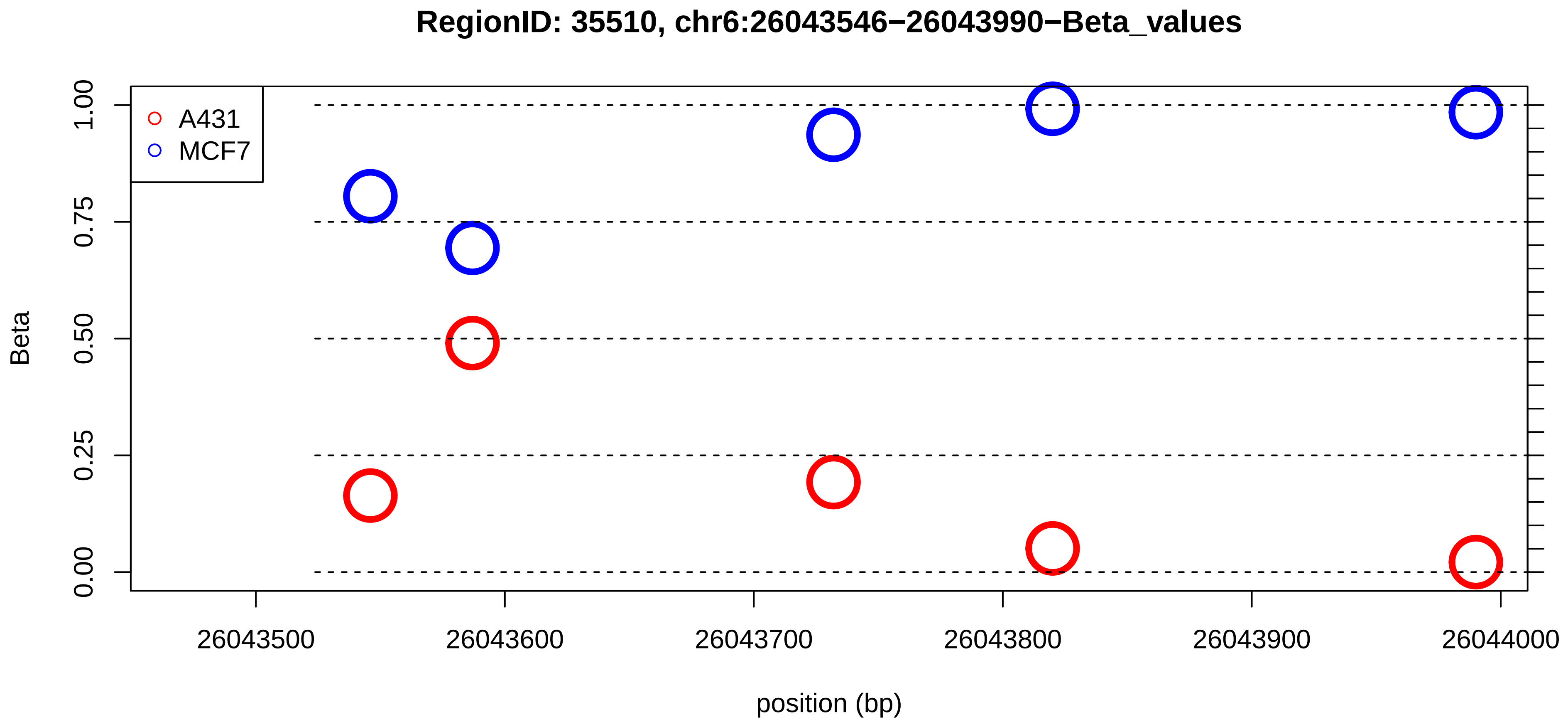
RegionID: 35510, chr6:26043546−26043990−Beta_values
A431
MCF7
Beta
1.00
0.75
0.50
0.25
0.00
26043500
26043600
26043700
26043800
26043900
26044000
position (bp)

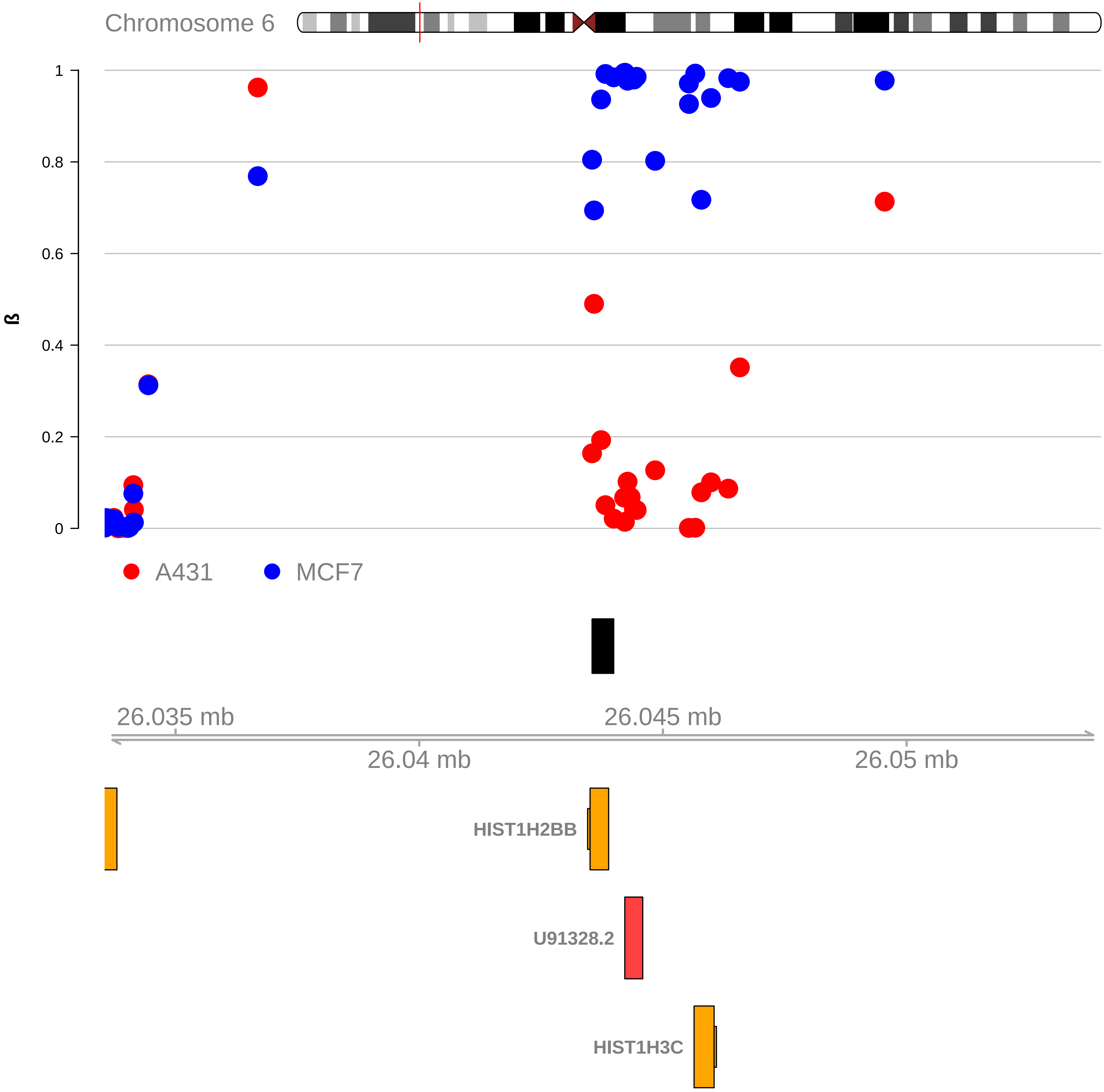

Chromosome 6
1
0.8
0.6
0.4
0.2
0
ß
A431
MCF7
26.035 mb
26.045 mb
26.04 mb
26.05 mb
HIST1H2BB
U91328.2
HIST1H3C
